# A Fusion Model for Intelligent Diagnosis of Gear Faults with Small Sample Sizes

**DOI:** 10.3390/s25175230

**Published:** 2025-08-22

**Authors:** Jianing Huang, Zikang Liu, Jianggui Han, Chenghao Cao, Xiaofeng Li

**Affiliations:** 1 College of Power Engineering, Naval University of Engineering, Wuhan 430072, China; 1920191014@nue.edu.cn (J.H.);; 2 Zhejiang University-University of Illinois Urbana-Champaign Institute, Zhejiang University, Haining 314400, China

**Keywords:** fusion model, gear fault, intelligent diagnosis, small sample size

## Abstract

Gear faults are a frequent cause of rotating machinery breakdowns. There are two open issues in the current intelligent diagnosis model of gear faults. (1) Shallow models demand fewer data but necessitate feature extraction from raw signals, relying on prior knowledge. (2) Deep networks can adaptively extract fault features but require large datasets to train hyperparameters. In this paper, a novel fusion model, called CBAM-TCN-SVM, is proposed for intelligent gear fault diagnosis. It consists of a temporal convolutional network module (TCN), a convolutional block attention module (CBAM), and a support vector machine (SVM) module. More specifically, the frequency-domain sequence data are fed into the CBAM-TCN model, which effectively extracts deep fault features via multiple convolutional layers, channel attention mechanisms, and spatial attention mechanisms. Then, the SVM classifier is employed for intelligent classification. The fusion model combines the advantages of deep networks and shallow classifiers, addressing the issues that arise when the accuracy of fault diagnoses is constrained by the data scale and feature extractions rely on prior knowledge. The experiments result in the proposed method achieving a classification accuracy of 98.3% and demonstrate that it is a feasible approach for predicting gear faults.

## 1. Introduction

Rotating machinery is a cornerstone of modern industrial systems, in which the gears serve as the pivotal components responsible for transmitting motion and power within these configurations. The reliable operation of gears is crucial, as gear faults are among the most prevalent causes of breakdowns for rotating machinery. The faults, if not promptly identified and addressed, can lead to extensive damage, potential safety hazards, and significant financial loss [1,2,3]. Consequently, the development of intelligent gear fault detection and diagnosis methods has emerged as a critical area of research, particularly for methods that leverage advanced deep learning algorithms without the need for equipment disassembly [4].

In the realm of gear fault diagnosis, diagnostic techniques by shallow models and deep network models have been widely explored [5,6]. The shallow models, such as support vector machines (SVMs) and decision trees, are known for their relatively low data requirements [7]. These models can perform well with limited training samples, making them suitable for scenarios where data acquisition is challenging or expensive [8]. However, a major drawback of shallow models lies in their reliance on feature extractions from raw signals, a process that heavily depends on prior knowledge of the system and the nature of the faults [9]. For example, Moshrefzadeh [10] proposed a new spectral amplitude modulation technique to isolate signaling components across varying energy levels, independent of load and speed conditions. By analyzing the envelope spectrum impulses of these extracted signals, the operational smoothness could be quantified. These metrics serve as inputs for machine learning algorithms, enabling intelligent bearing fault diagnoses. The researchers had to carefully select and extract relevant features from the raw vibration signals, such as time-domain statistics and frequency-domain spectral features, to achieve an acceptable diagnostic accuracy. This manual feature extraction process not only demands significant time and effort but also limits the model’s generalizability to new and unseen fault patterns [11].

The deep network models, including convolutional neural networks (CNNs) [12], recurrent neural networks (RNNs) [13], and temporal convolutional networks (TCNs) [14], have gained popularity due to their ability to adaptively extract fault features from raw data. These models can automatically learn hierarchical representations of the input data, capturing complex patterns and relationships that may be difficult to identify through manual feature engineering. For example, Yuan et al. [15] developed a sophisticated fault diagnosis framework grounded in big data and deep learning. They trained CNN models on diverse signals, including vibration, voltage, current, and acoustic data, enabling high-accuracy fault detection and wear prediction across more than ten types of rotating machinery, such as rolling bearings and gearboxes. The deep network models can directly process vibration signals and learn meaningful features through multiple convolutional and pooling layers, achieving high diagnostic accuracies. However, deep networks typically require large datasets to train the numerous hyperparameters within the network structure [16]. Insufficient training data can lead to overfitting, where the model performs well on the training data but fails to generalize to new, unseen data [17]. Moreover, training deep networks can be computationally expensive and time-consuming, especially when dealing with high-dimensional data.

Current research in fault diagnosis with limited data samples primarily addresses data scarcity, class imbalance, and noisy or variable operating conditions through synergistic strategies. For instance, Li et al. [18] proposed auxiliary generative mutual adversarial networks (AGMANs) with dual discriminators to mitigate class imbalance, improving gearbox diagnosis accuracy by 12% under the small samples. Similarly, Liu et al. [19] integrated CNN-LSTM-GAN to handle biased hydropower data, boosting fault recall by 160%. Transfer learning leverages pretrained knowledge for new domains. Zhang et al. [20] utilized LSTM networks pretrained on normal operational data, reducing prediction errors by 22% with only 10 fault samples in electric drive systems. Additionally, attention-based hybrid models improve noise robustness. Ma et al. [21] combined multiscale depthwise separable convolution, bidirectional GRU, and squeeze-excitation mechanisms to attain 94.3% accuracy for gearbox diagnosis under 10 dB noise. Physics-informed methods embed mechanical constraints into deep learning. Sun et al. estimated fault severity with 95.4% accuracy using only one real sample, by compensating for transfer function discrepancies via gear dynamics equations [22]. Benefiting from previous valuable works, significant contributions to the service reliability of bearings, gears, etc., have been made. However, challenges persist in computational efficiency and cross-domain adaptability in fault diagnosis with a limited data sample.

Given the limitations of both the shallow and deep models, the development of hybrid models, which can combine the advantages of the different approaches, has become an active area of research. Hybrid models aim to leverage the low data requirements of shallow models and the adaptive feature extraction capabilities of deep networks to achieve better fault diagnosis results [23]. One such approach is to use a deep network for feature extraction and a shallow classifier for final classification. For example, Ding et al. [24] proposed a gearbox fault intelligent recognition method that combines a TCN with a soft thresholding algorithm (SAM-TCNST). The TCN is used to extract features from raw vibration signals, and a soft thresholding algorithm is applied to denoise the features. However, this method still requires preprocessing of the raw vibration signals, and fails to achieve an end-to-end gearbox fault diagnosis.

Another aspect that needs to be considered in gear fault diagnosis is the choice of classifier. The traditional TCNs used for fault diagnosis often employ Softmax classifiers for fault classification. While Softmax classifiers can effectively integrate with the network during training and facilitate weight updates in TCN models, they have several drawbacks. They may perform poorly in nonlinear problems, have insufficient model generalizability, and be susceptible to overfitting, especially when dealing with small sample sizes [25,26,27]. In contrast, SVMs are known for their excellent performance in handling small sample sizes and nonlinear problems. SVMs can find the optimal hyperplane that maximally separates different classes in the feature space, exhibiting stronger generalization capabilities [28,29,30,31]. Therefore, replacing the Softmax classifier with an SVM classifier in a hybrid model can potentially improve the diagnostic accuracy and robustness of the system.

In this paper, we propose a novel fusion model, called CBAM-TCN-SVM, for intelligent gear fault diagnoses with small sample sizes. The model combines the advantages of different components to address the limitations of the existing approaches. First, fast Fourier transform (FFT) is used as a preprocessing step to transform the raw time-domain data of gear faults into the frequency domain, reducing noise and highlighting the key frequency components. The frequency-domain sequence data are subsequently fed into the CBAM-TCN model. The TCN module consists of multiple convolutional layers that can adaptively extract deep fault features from the input data. The CBAM module is incorporated into the TCN to enhance its feature extraction capabilities. CBAM enables the model to selectively focus on channels with key information, particularly in regions with significant signal morphology changes, highlighting the target detail information and reducing the redundant information. Finally, to further enhance the network’s classification capabilities, the SVM classifier is employed for intelligent classification.

The proposed fusion model addresses the two open issues in the current gear fault intelligent diagnosis model. The model combines the low data requirements of shallow models and the adaptive feature extraction capabilities of deep networks, reducing the dependence on prior knowledge for feature extractions and improving the diagnostic accuracy, even with small sample sizes. The experimental results demonstrate that the proposed method achieves a classification accuracy of 98.3%, providing a feasible approach for predicting gear faults. In summary, the main contributions of this paper are threefold.

1. The integration of CBAM into the TCN allows the fusion model to adaptively focus on channels with critical information, thereby enhancing the feature extractions and reducing the reliance on prior knowledge for manual feature engineering.
2. The proposed hybrid model leverages SVM’s superior performance in handling small sample sizes, which improves the model’s generalizability and robustness when dealing with limited training data, enhancing the overall diagnostic accuracy.
3. The fusion model addresses the limitations of both the shallow and deep models, providing a more effective and efficient approach for intelligent gear fault diagnosis, especially in scenarios with small sample sizes.

The remainder of our paper is organized as follows. Section 2 explains the theoretical background of the CBAM, TCN, SVM, and our fusion model. Section 3 introduces the experimental platform and analyzes the vibration data. Section 4 describes the comparative fault diagnosis results. Section 5 summarizes the methods presented in this paper.

## 2. Theory and Modeling

Feature extraction via the convolutional block attention module and temporal convolutional network (CBAM-TCN) is an advanced method for capturing complex fault patterns. It merges the TCN’s hierarchical, adaptive feature learning over time (handling short-and long-term temporal dependencies for gear faults) with the CBAM’s attention-enhancing mechanism. The CBAM, with channel and spatial attention, selects key channels for gear faults by weighting them, and focuses on spatial locations with significant signal changes. This combination enables the CBAM-TCN to extract deep, discriminative features, reducing manual feature engineering and boosting the gear fault diagnosis performance.

### 2.1. Basic Theory of the TCN

A TCN is a variant of a convolutional neural network (CNN) specifically designed for sequential tasks. Its core idea is to effectively enhance the network’s ability to process time series information by introducing causal convolutions and dilated convolutions, as well as using residual connections to construct CNNs. In the TCN, the causal convolutions, dilated convolutions, and residual connections are included. In the causal convolutions section, the design of the TCN must obey the following rules: first, the output data must be causal in the time dimension; then, the data scale must remain unchanged. To ensure causality, only neurons at time t and earlier are used to compute the neuron at time t in the second layer. When gradually computing neurons closer to time 0 in the next layer, there may be insufficient neurons in the previous layer [25]. To maintain an unchanged sequence length, zero padding is performed on the left side. Currently, causal convolutions have a problem. To expand the receptive field, the convolution kernel dimension or the number of layers needs to be increased, which increases the network’s complexity and training difficulty.

Using dilated convolutions can solve this problem by skipping some elements during the convolution, which expands the receptive field without increasing the network’s depth or the number of parameters. The dilated convolution function *F*(*x*) for an input sequence *x* and convolution kernel *C* can be represented by Formula (1):

(1) $$F(x)=\sum_{i=0}^{k-1}C(i)x_{s-di}$$

where *k* is the convolution kernel size, *i* is the position of the convolution kernel, *d* is the dilation factor, and *s−di* represents the element of the previous convolutional layer.

To solve the vanishing and exploding gradient problems often encountered in deep network training, residual connections are introduced. Residual connections solve the problem of gradient instability in temporal convolutional networks by adding a direct pathway between convolutional layers. The calculation method involves adding the network’s output *F*(*x*) to the input x and then passing it through an activation function to obtain the output P, as shown in Formula (2):

(2) $$P=A_{cti}(x+F(x))$$

**Figure 1:**
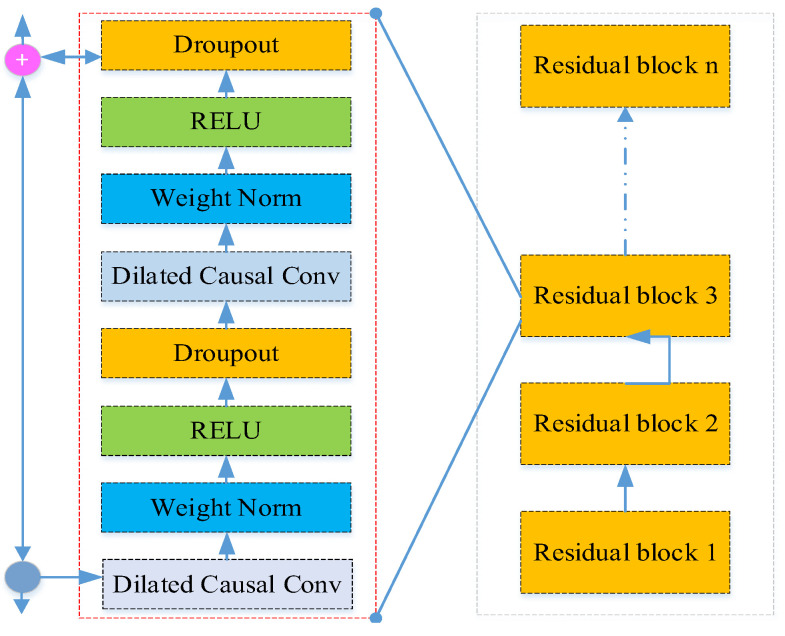
Structures of residual blocks in the TCN.

*A* residual block consists of dilated causal convolutions, weight normalization, ReLU activation functions, and dropouts. The TCN network is composed of many stacked residual blocks, as shown in Figure 1, which illustrates a TCN network with n stacked residual blocks. Each residual block has a convolution kernel size of k and a dilation factor of 2n − 1 in the n-th residual block.

### 2.2. Basic Theory of CBAM

**Figure 2:**
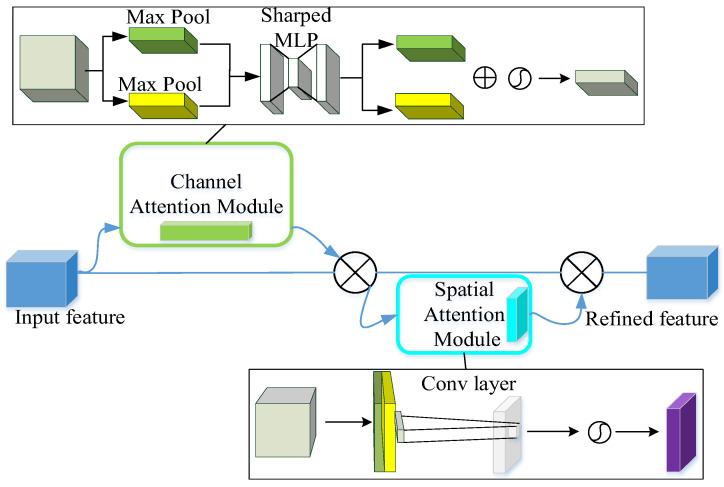
Overall architecture of CBAM.

CBAM is a simple yet effective attention mechanism applicable to feedforward convolutional neural networks. It innovatively designs a channel attention module and a spatial attention module to emphasize meaningful features along the channel axis and spatial axis, respectively. The overall architecture of CBAM is shown in Figure 2:

CBAM models the relationships between different channels to learn the important weights of each channel while also focusing on the spatial dimension by learning the weights of each pixel position to emphasize important regions in the feature map. Introducing a CBAM layer after the TCN layer enables the TCN to focus more on the important features in the feature map, improving the network’s expressive capabilities and performance, which is beneficial for enhancing the accuracy and robustness of fault diagnoses.

### 2.3. Support Vector Machine

SVM stands as a statistical-theory-driven machine learning approach, adept at tackling classification challenges. It has gained extensive traction in the field of nonlinear and high-dimensional pattern recognition tasks. In gear mechanisms, a prevalent issue is the scarcity of collected data, which hampers the acquisition of sufficient fault samples and yields a constrained dataset [32]. Given this, SVM is frequently employed for diagnosing gear faults. Its core mechanism involves transforming the initial nonlinear problem from a low-dimensional input space into a high-dimensional feature space to derive a viable solution, with the optimal function formulated accordingly:

(3) $$f(x)=\mathrm{sgn}(\sum_{i=1}^{N}\alpha_{i}*y_{i}K(x_{i},x)+b^{*})$$

Typical kernel functions encompass linear, polynomial, radial basis function (RBF), and sigmoid kernels. For tackling nonlinear classification tasks, the RBF kernel offers distinct benefits, including its capacity to project samples nonlinearly into a higher-dimensional space and its leniency regarding numerical constraints. Hence, this study opts for the RBF kernel, with the resultant decision classification function formulated as below:

(4) $$f(x)=\mathrm{sgn}(\sum_{i=1}^{N}\alpha_{i}*y_{i}\exp(\frac{-{\left\|x-x_{i}\right\|}^{2}}{2g^{2}})+b^{*})$$

In the formula, *g* denotes the kernel parameter governing the scope of the kernel function’s influence. The SVM’s classification efficacy hinges on the choice of pivotal parameters, with the penalty parameter *C* and kernel parameter g exerting a profound effect on classification precision and generalization performance. The penalty parameter *C* balances the reduction of model complexity against the minimization of empirical risk, while the kernel parameter *g* influences the correlation intensity among support vectors. Excessively large values may cause overfitting to training data, whereas overly small values may limit flexibility. Parameter optimization is performed using a grid search. All possible parameter combinations are generated within a predefined parameter space, and k-fold cross-validation is employed to evaluate the model performance of each combination. After traversing all parameter combinations, the (*C*, *g*) with the highest cross-validation score is selected as the optimal parameter.

### 2.4. Model Establishment

**Figure 3:**
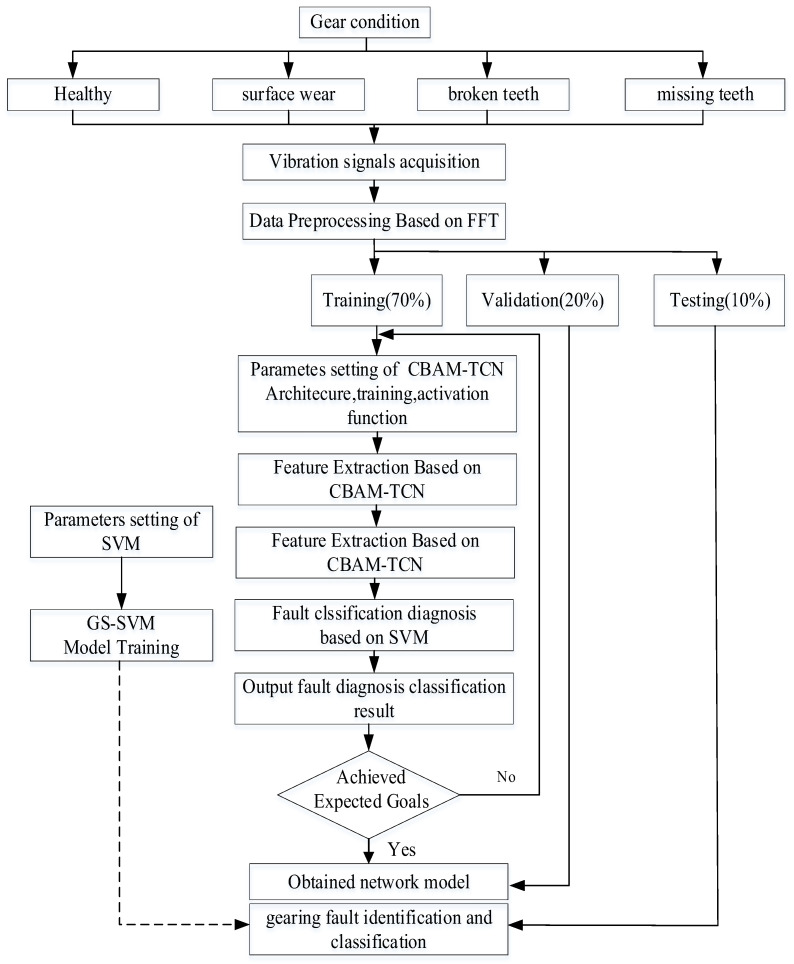
The hybrid model fault diagnosis process.

The proposed FFT-CBAM-TCN-SVM hybrid model fault diagnosis process is shown in Figure 3. The model’s overall structure comprises three key stages: fault signal preprocessing, feature extraction, and fault diagnosis. During fault signal preprocessing, vibration signals are gathered from bearings, and sample lengths are defined. Each sample undergoes standardization to enhance the integrity and dependability of the extracted fault data. FFT is employed for data preprocessing, after which the dataset is divided into training, validation, and test sets based on a predetermined ratio. In the feature extraction phase, the processed data are fed into the CBAM-TCN model for feature mining. This study incorporates two CBAM-TCN residual blocks, with 64 convolution kernels, a kernel size of 10, and dilation factors of 1 and 2. Each residual block applies weight normalization post-convolution, utilizing the ReLU activation function. The dropout rate is configured to 0.005, and the learning rate is set at 0.001.

The extracted temporal features are fed into an SVM for training and classification. As indicated by the dashed line in Figure 3, the CBAM-TCN and grid search parameter optimization components undergo independent stagewise training. First, the CBAM-TCN network is trained end-to-end using backpropagation to learn hierarchical representations of frequency-domain features. Subsequently, with the CBAM-TCN parameters frozen, the extracted features are input to the SVM classifier, where hyperparameter optimization is performed via grid search. This approach circumvents gradient interference during joint backpropagation, thus enhancing training stability under small-sample scenarios.

## 3. Testing Platform

**Figure 4:**
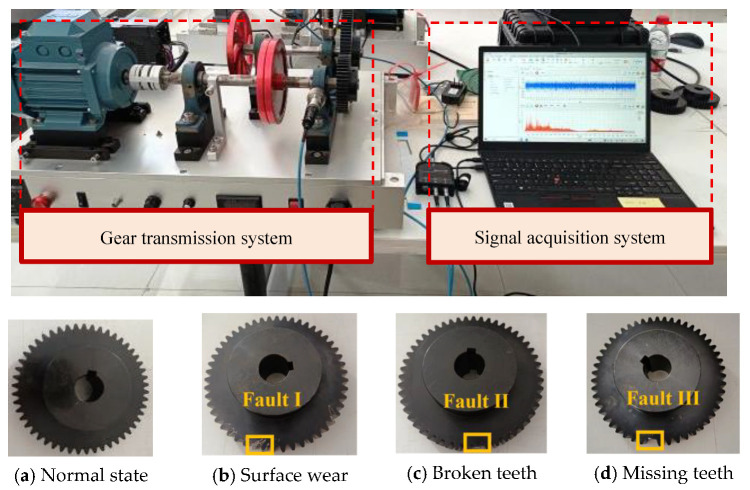
Gear fault test rig with different fault conditions.

This study focuses on the diagnosis of three common mechanical faults of industrial gears: surface wear (Fault I), broken teeth (Fault II), and missing teeth (Fault III). To verify the validity of the hybrid model proposed in this paper, a gear transmission platform and the gear faults are established in the laboratory, as shown in Figure 4.

**Figure 5:**
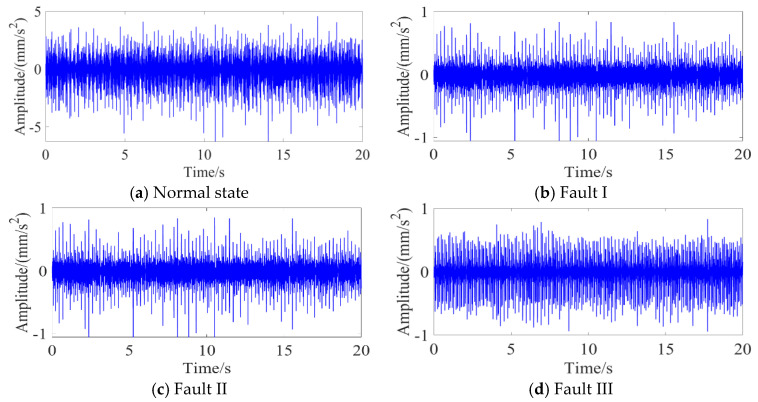
Original vibration waveforms of the four conditions.

The experimental platform comprises two primary systems, a mechanical transmission system and a signal acquisition system. The mechanical transmission system integrates a Siemens three-phase AC motor, programmable controller, frequency converter, gear transmission mechanism, and couplings. To obtain high-fidelity vibration data, the signal acquisition system employs two low-noise accelerometers (AC500-2P) with a ±80 g peak measurement range and 100 mV/g sensitivity. These sensors are strategically mounted on both the horizontal and vertical axes of the gear transmission mechanism’s support bearings. Vibration data acquisition is performed via a GX400 four-channel data recorder at a sampling frequency of 48 kHz. Four operational conditions are investigated: normal operation and three fault modes (missing tooth, broken tooth, and surface wear). Vibration signals under these conditions are systematically collected, resulting in four distinct datasets. As illustrated in Figure 5, the vibrations in the time domain are presented for a comparative analysis across the four operational states.

**Figure 6:**
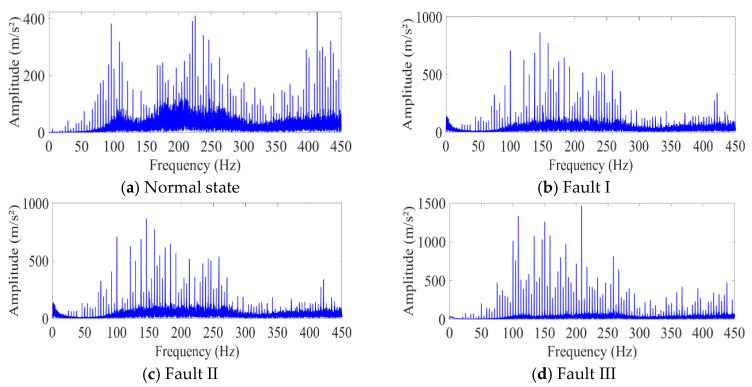
Frequency spectra of the four conditions.

For the analysis, the frequency spectra of the four conditions are further given in Figure 6. In the normal state, the frequency spectrum is dominated by the fundamental frequencies related to the gear’s rotational speed and meshing frequency, with relatively low-amplitude harmonics. For Fault I, there are slight increases in the amplitudes of certain frequency components compared with those in the normal state, possibly due to the additional vibrations generated by surface wear. These increased amplitudes may be associated with the excitation of new resonant frequencies or the modulation of existing frequencies. Fault II results in more significant changes in the frequency spectrum. New frequency components appear, which are likely related to the impact forces caused by the broken tooth. These additional frequencies can be attributed to the transient vibrations generated when the broken tooth engages with other teeth during rotation. Fault III also shows distinct changes in the frequency spectrum. There are large-amplitude spikes at specific frequencies, which are a clear indication of the severe impact of the missing tooth on the gear’s vibration behavior. These spikes are likely associated with sudden changes in gear motion and the resulting dynamic forces. Overall, the frequency spectra provide valuable information about the different fault conditions, and the proposed fusion model can leverage these spectral features to accurately classify the gear faults, especially in scenarios with small sample sizes, as demonstrated in the experimental results.

## 4. Results and Analysis

To validate the effectiveness and diagnostic performance of the proposed FFT-CBAM-TCN-SVM methodology, four experimental datasets representing distinct operational states (the normal conditions and three fault modes: missing teeth, broken teeth, and surface wear) are utilized. Each operational state contains 300 experimental samples, with each sample comprising 6000 data points. The datasets are partitioned into training (70%) and validation (30%) subsets for systematic model development.

### 4.1. Model Analysis

**Figure 7:**
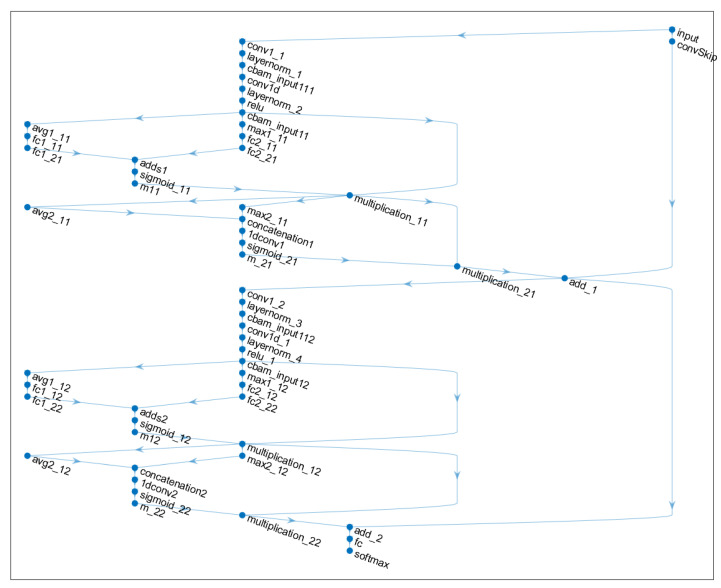
Network structure diagram of CBAM-TCN.

The TCN model incorporates the Adam optimizer to enable adaptive learning rate adjustments and parameter updates along the negative gradient direction, thereby accelerating network convergence. The leaky ReLU activation function is selected to enhance the nonlinear feature extraction capability. The maximum number of training iterations is set to 50. Table 1 and Table 2 provide detailed hyperparameter settings for the TCN model and the GS-SVM model, respectively. The initial value for the optimal penalty parameters of C is set to 1, and the initial value for the nuclear parameters of g is set to 0.1. The article sets up two CBAM-TCN modules, and the network structure is shown in Figure 7.

### 4.2. Experimental Results and Analysis

After the architecture of the CBAM-TCN model is finalized, the TCN is integrated with a support vector machine (SVM) classifier. To mitigate computational resource requirements, the SVM uses a radial basis function (RBF) kernel. For a comparative performance analysis of the different hybrid models and enhanced experimental reliability, each methodology undergoes 10 independent trials, with the mean values of repeated experiments serving as evaluation metrics. As shown in Table 3, multiple comparative experiments with other common classifiers are conducted to verify the capability of the fusion model for gear fault diagnosis.

**Figure 8:**
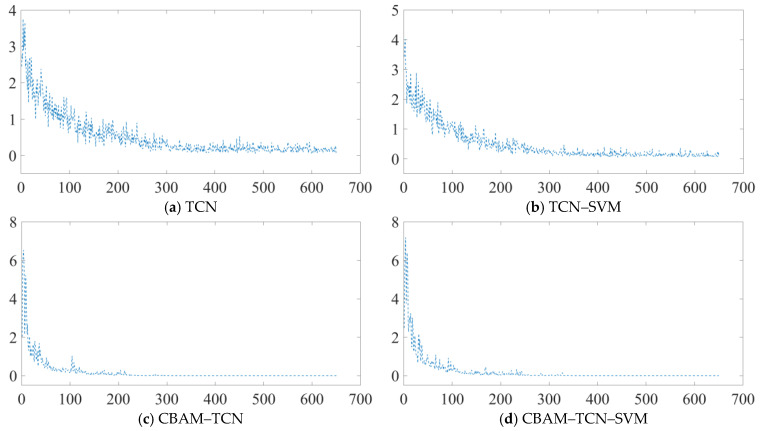
Model training loss curve.

Among the traditional methods, radial basis function networks (RBFs), extreme learning machines (ELMs), random forests (RFs), and SVMs exhibit relatively low and stable accuracy levels, with values of −0.4. The introduction of deep learning methods represents a significant improvement. The CNN demonstrates a notable increase in accuracy, with the maximum accuracy reaching approximately 0.85. The hybrid models CNN-SVM and TCN-SVM also show promising results, suggesting that combining deep learning models with SVM can enhance the overall performance to some extent. The TCN stands out for its high accuracy, where the minimum, maximum, and average accuracies are relatively high, approaching 0.95; this highlights the TCN’s strong ability to handle the given data, which is likely due to its effectiveness in processing sequential or time series information. The proposed CBAM-TCN-SVM model achieves the best performance among all the methods. Both the minimum and maximum accuracy bars are the highest, and the average accuracy line approaches 1. This outstanding performance can be attributed to the integration of the convolutional block attention module (CBAM). The attention mechanism in CBAM enables the model to focus more on important features, thereby significantly improving the accuracy. In conclusion, the proposed CBAM-TCN-SVM model is superior to the other methods in terms of accuracy. Figure 8 further reveals the training dynamics, showing gradual stabilization and corresponding accuracy improvements for all the TCN variants during the iterative optimization. This observation underscores the inherent stability of TCN-based frameworks for fault detection, thereby validating the scientific importance of enhancing this foundational architecture.

Increased fluctuations in early-stage classification accuracies following CBAM integration can be detected. This phenomenon stems from the attention mechanism’s design objective to mitigate the computational overhead associated with sliding-window-based exhaustive search strategies. During the initial training phases, insufficient feature knowledge accumulation may lead to signal misinterpretations in critical regions, potentially resulting in false negatives. However, as evidenced in the latter training stages, the stability of classification accuracy—particularly when attention-based training strategies are employed—demonstrates marked post-convergence enhancements.

In addition to classification accuracy comparisons, Table 4 provides a comprehensive evaluation of the four TCN architectures through metrics including training duration, F1 score, and recall rate, enabling multidimensional performance assessment.

As shown in Table 2, the CBAM-TCN-SVM architecture demonstrates superior performance across multiple evaluation metrics. Specifically, accuracy improvements of 7.6%, 6.7%, and 1.7% are observed compared with those of the baseline models, indicating an optimal comprehensive performance. The recall rate reaches 0.984, reflecting enhancements of 6.8%, 6.3%, and 1.6%, respectively, which signifies an improved true positive recognition capability with reduced false-negative occurrences. The F1 score of 0.982 (representing 6.6%, 6.3%, and 1.9% improvements) demonstrates an effective balance between precision and recall, ensuring robust positive sample identification while minimizing false-positive errors.

However, the integration of the CBAM module into the TCN architecture resulted in a 37.31 s increase in training duration. This computational trade-off is attributable to the attention mechanism’s feature refinement process that makes use of channelwise nonlinear transformations. Considering the substantial performance gains achieved, the additional computational cost associated with CBAM implementation is deemed acceptable to obtain the enhanced diagnostic capabilities. Furthermore, the training duration of the CBAM-TCN-SVM architecture is shorter than that of the CBAM-TCN model. This discrepancy arises from the decoupled training paradigm, where the TCN component focuses on feature learning while the SVM handles classification decision-making, thereby reducing end-to-end parameter optimization complexity. In contrast, conventional TCN architectures require a simultaneous optimization of all the network parameters during backpropagation, leading to exponential computational growth with increased input sequence lengths or network depths.

**Figure 9:**
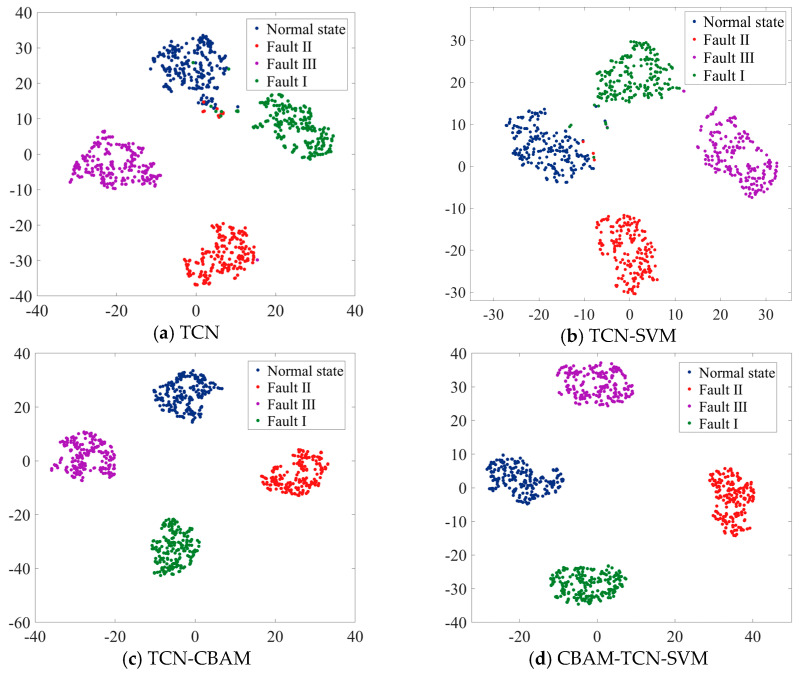
Fault characteristics before and after model recognition.

Notably, the observed training time differences pertain specifically to model calibration rather than operational inference. For practical mechanical fault diagnosis applications, the processing time during real-time deployment is more important than the training duration, particularly given that fault events typically develop over extended periods. The 98.3% classification accuracy achieved by the proposed methodology indicates a state-of-the-art performance in AI-based fault recognition, demonstrating clear superiority over comparative architectures. To facilitate an intuitive interpretation of the model’s feature learning capabilities and classification decision boundaries, t-distributed stochastic neighbor embedding (t-SNE) dimensionality reduction is applied to visualize the fault classification process on the test dataset, as illustrated in Figure 9.

In Figure 9a, the data points of different states are somewhat clustered but with significant overlaps among them; this indicates that the TCN model alone has a certain ability to distinguish between different states, but the separation is not distinct enough, which may lead to relatively high misclassification rates. Figure 9b shows a slightly better separation than Figure 9a. The SVM component seems to enhance the model’s ability to define boundaries between the different state clusters. However, there are still some overlapping regions, especially between certain fault states, suggesting that while the combination improves performance, it is not yet optimal. Figure 9c shows a more refined clustering of data points. The addition of the CBAM helps the model focus on more relevant features, resulting in tighter and more distinct clusters for each state. The overlaps among the different state clusters are reduced compared with those of the previous two models, indicating an improvement in the model’s discriminative ability. Figure 9d shows the best performance among the four methods. The data points for each state form well-separated and compact clusters. The combination of CBAM, TCN, and SVM effectively leverages the strengths of each component. The attention mechanism in CBAM helps the model extract more informative features, the TCN processes the sequential information well, and the SVM establishes clear decision boundaries. This model has the greatest potential for accurately classifying different states with minimal misclassifications.

In summary, the aforementioned experimental evaluation substantiates the robust diagnostic capability of the developed CBAM-TCN-SVM methodology, demonstrating high-precision fault identification across various gear failure modes. These findings conclusively validate the practical feasibility of the proposed framework for industrial gearbox fault diagnosis applications.

### 4.3. Ablation Experiments

To systematically evaluate the contribution of TCN (temporal convolutional network), CBAM (convolutional block attention module), and SVM (support vector machine) in the hybrid model, we designed five groups of ablation experiments to verify the effectiveness of their integration. As shown in Table 5, the experimental parameters remain consistent across all configurations. By adding or removing core components using the control variable method, we compare the classification performance of the models on the small-sample gear fault dataset.

In the exclusive implementation of SVM (Group 1), the conspicuously low accuracy and F1 score, attributable to the absence of temporal dependency and feature correlation exploration, underscore the inefficacy of conventional classifiers in small-sample temporal diagnostic paradigms. The integration of TCN (Group 2), which harnesses causal convolution to model temporal dependencies, elevates the accuracy to 0.908, thereby validating its pivotal role as a cornerstone for temporal fault information extraction. A comparative analysis between TCN alone (Group 2) and the TCN + SVM ensemble (Group 3) reveals a discernible performance improvement, stemming from SVM’s enhancement of fault boundary delineation. In contrast, the TCN + CBAM configuration (Group 4) demonstrates a substantial performance boost: CBAM suppresses extraneous features and accentuates fault-sensitive regions, thereby augmenting feature discriminability. The fully integrated CBAM-TCN-SVM architecture (Group 5) achieves optimal efficacy. Through the synergistic interplay of TCN’s temporal pattern mining, CBAM’s feature refinement, and SVM’s boundary reinforcement, the model effectively mitigates challenges intrinsic to small-sample scenarios, including feature noise, information sparsity, and overfitting proclivity. This culminates in a high-fidelity diagnostic framework, offering a robust solution for industrial fault diagnosis under data-constrained conditions.

## 5. Conclusions

In conclusion, this study introduces a novel CBAM-TCN-SVM fusion model for intelligent gear fault diagnosis with small sample sizes. Through comprehensive experiments, the model demonstrated its superiority, with a high classification accuracy of 98.3%. The integration of CBAM enhances feature extraction by focusing on critical information, whereas the SVM classifier improves nonlinear pattern recognition, especially under limited-data conditions. This model effectively addresses the limitations of both the traditional shallow and deep models, providing a practical and efficient solution for industrial gearbox fault diagnoses. While this study focuses on gear fault diagnoses under constant-speed operating conditions, real-world industrial scenarios often involve variable speed regimes. In the future, further investigations are warranted to validate the proposed model’s performance under speed-varying conditions. Moreover, incorporating data augmentation techniques to further enhance the robustness of small-sample diagnosis will be further explored, aiming to provide a more comprehensive solution reference for this field.

## Figures and Tables

**Table 1:** TCN model hyperparameter settings.

Model Hyperparameter	Parameter Setting	Model Hyperparameter	Parameter Setting
Optimization algorithm	Adam	Activation function	Leaky ReLU
Loss function	Categorical entropy	Expansion factor	1, 1
Convolution kernel	64	Convolution kernel size	10
Random inactivation factor	0.1	Learning rate	0.001

**Table 2:** GS-SVM model hyperparameter settings.

Parameter Setting	Values/Range	Optimization Result/Objective
Penalty Factor C	C ∈ [2^−5^, 2^10^]	Best c: 0.031
Kernel Parameter g	g ∈ [2^−5^, 2^10^]	Best g: 0.250
Cross-Validation Folds	5	Evaluates generalization capability
Kernel Function Type	RBF	Adapts to data linear separability

**Table 3:** Accuracy comparisons of different methods.

Model Name	Accuracy	Precision	Recall	F1 Score
BP	33.2%	0.331	0.332	0.333
RBF	35.2%	0.351	0.353	0.352
ELM	36.4%	0.366	0.365	0.364
RF	32.8%	0.330	0.326	0.328
SVM	33.6%	0.329	0.336	0.332
CNN	83.3%	0.840	0.839	0.840
CNN-SVM	85.0%	0.868	0.855	0.861
CNN-LSTM	52.5%	0.518	0.525	0.521
MCNN-BiGRU-Attention	78.8%	0.823	0.788	0.805
TCN	90.8%	0.916	0.916	0.916
TCN-SVM	91.6%	0.917	0.921	0.919
CBAM-TCN	96.6%	0.958	0.968	0.963
CBAM-TCN-SVM	98.3%	0.980	0.984	0.982

**Table 4:** Four model evaluation indicators.

Evaluation Index	TCN	TCN-SVM	CBAM-TCN	CBAM-TCN-SVM
Training time	64.251	63.965	101.275	97.956
F1 score	0.916	0.919	0.963	0.982
Recall	0.916	0.921	0.968	0.984
Accuracy rate	90.8%	91.6%	96.6%	98.3%

**Table 5:** Four model evaluation indicators.

Number	TCN	CBAM	SVM	Accuracy	Precision	Recall	F1 Score
1	×	×	√	0.336	0.332	0.338	0.335
2	√	×	×	0.908	0.916	0.916	0.916
3	√	×	√	0.916	0.917	0.921	0.919
4	√	√	×	0.966	0.958	0.968	0.963
5	√	√	√	0.983	0.980	0.984	0.982

## Data Availability

The data that support the findings of this study are available from the corresponding author upon reasonable request.
